# The Prophylaxis of Venereal Diseases

**Published:** 1868-07-15

**Authors:** F. J. Bumstead

**Affiliations:** Professor of Venereal Diseases at the College of Physicians and Surgeons, N.Y.


### THE PROPHYLAXIS OF VENEREAL DISEASES.

BY F. J. BUMSTEAD, M.D., PROFESSOR OF VENEREAL DISEASES AT THE COLLEGE OF PHYSICIANS AND SURGEONS, N.Y.

"*Cito, tuto, . . . et jucunde.*"

But little is said of the prophylaxis of venereal diseases in English or American treatises on venereal. Our Anglo-

Saxon morality is so high-toned that we shrink from placing any obstacle in the way of vice meeting with its just reward, and so inconsistent that we are glad to shorten the misery once incurred and—pocket the fee! But when we reflect that the passions always will control, as they always have controlled, the moral sense of the greater part of man and womankind, and that the effects of vice are by no means confined to the guilty, it is evident that this subject is not unworthy of consideration.

The confession of one's faults is regarded as a virtue, and yet it has always appeared to me that if anything could aggravate the injury done by an unfaithful husband to his wife, it was the revelation of his guilt, to be brooded over by her in many a lonely hour, perhaps forgiven but never forgotten; and the demands of the *ménage* may be imperative, if the secret is not to be divulged.

The danger in coitus may be said to be two-fold: 1st, That of contracting gonorrhœa; 2nd, That of contracting either a chancroid or chancre. This division should be borne in mind in the consideration of prophylaxis, because the first mentioned disease has its seat either within the urethra or vagina, while the external parts alone are commonly exposed to the latter; and although certain precautions are equally applicable for both, the same is not true of all. Moreover, precautionary measures must vary somewhat with the opposite sexes, and also according as it is the object either to avoid contracting or avoid giving disease.

Gonorrhœa originates in two ways: 1st, By direct contagion; 2nd, By any source of irritation acting upon the urethra, such as leucorrhœal discharge, especially if aggravated by repeated or prolonged intercourse, unusual sexual excitement, violent exercise, etc. The latter mode of origin is even more frequent than the former.

Direct contagion from the gonorrhœal virus in both sexes is best avoided by the use of an article—the condom—which, tradition says, was invented by an Englishman visiting Paris, and was called after his name, but which gained for him such unenviable notoriety that he was obliged to change his appellation. Madame de Stael is said to have pronounced it "a cuirasse against pleasure, a cobweb against danger;" but the poor lady must have been unfortunate in her selection of the article, which is still justly regarded as affording the best protection within the power of art. Condoms made of the cæcum of the sheep are most reliable; those of vulcanized rubber, especially when old, are brittle and liable to break.

In the absence of any such protective covering, the greatest safety for the male who is is exposed, consists in urinating directly after the act of coitus, and, once or twice, as the urine is flowing, in compressing the lips of the meatus, so as to distend the fossa navicularis with the fluid, which subsequently escapes with some force, and washes out any purulent matter which may have gained entrance within the meatus. As the Latin adage has it:

> "Post coitum si mingas,

> Apte servabis urethras."

For the female, emptying the bladder is equally desirable, and the vagina should be thoroughly and copiously washed out, by means of a Davidson's syringe and a pint or two of water.

Authorities differ as to the advisability of urethral injections as a prophylactic after suspicious intercourse. Diday recommends one of a weak solution of sulphate of zinc, and has invented for the purpose a minute syringe—a sort of gentleman's pocket companion, so small as to be carried in the vest pocket, or concealed in the palm of the hand, the shaft of the piston being furnished with a hinge, so that—the instrument having been filled before leaving home—it can be bent at a right angle, and the contents will not be forced out until the proper time for using it. Ricord, on the contrary, objects decidedly to the use of urethral injections, as likely to add to the irritation of the canal, and it is probable that a thorough cleaning with the urine, in the manner just indicated, is to be preferred.

In the case of a man or woman affected with a discharge, and in fear of communicating it to another, it is evident that these precautions—micturition and vaginal injections—should precede the act of coitus.

But the causes of gonorrhœa, aside from direct contagion, are also of great importance; and it can not too often be repeated, that a large proportion of claps are contracted under circumstances of especial excitement, and with more moderation might be avoided. Frequently, intercourse has already taken place with impunity between the same parties, but then comes a dinner, wine, a dance, a night of *orgie*, and —a clap! Ricord's prescription for catching a clap will also serve to indicate how to avoid it:

℞. "A woman of a lymphatic temperament, pale, a blonde rather than a brunette (the more white she has the better), invite her to dinner; order oysters for your first course, and asparagus for the second; drink often and freely—white wines, champagne, coffee, liqueurs, they are all excellent; after dinner dance a while, and make your companion dance with you; get well heated, both of you, and quench your thirst with beer; at night play your part valiantly, two or three times are not too much, and oftener would be better; in the morning, don't forget to take a warm bath, nor neglect to give yourself an injection.

"This programme having been conscientiously followed out, if you don't have the clap, it is because Providence has preserved you."

Again, women should be avoided a day or two before, and especially for a few days after, their menstrual periods, for it is particularly at these times that the muco-purulent secretion, from which the vagina of but few of the gentler sex at the present day can be said to be free, possesses irritant qualities sufficient to light up urethoitis in the male. It is a question whether the law of Moses, prohibiting intercourse within seven days after the menses, was not intended as a hygienic precaution.

We come now to the second class of diseases mentioned at the outset, viz.: the chancroid and true syphilis. As regards their prophylaxis, the condom is less efficacious than against gonorrhœa, at least when it is the woman who is diseased and the man who is exposed. As is well known, the labia are a favorite seat of mucous patches, the secretion of which is but little, if at all, less contagious than that of a primary lesion or chancre; and these parts, *in coitu*, are brought in contact with the pubes and the root of the penis, which a condom does not protect. I have at present under my care a young medical man, who finished his education in Paris, and while there indulged in some of the gaieties of that gay city. Thinking to protect himself from harm, he was in the habit of using a *capote*, which, however, did not prevent his contracting a chancre at the peno-scrotal angle. Ricord, to whom he first applied for treatment, reminded him of the folly of exposing himself to a rain storm with an umbrella over his head, but with his feet bare!

Any preliminary attention to cleanliness for a person exposed to contract these diseases is a mistake, for the natural sebaceous secretion of the parts affords a degree of protection that can only be less perfectly supplied by the use of sweet oil, simple cerate, and other unctious substances.

For a person already diseased and liable to convey the disease to another, previous cleanliness may be of some value in removing any collection of matter, and cauterization of the sores may also lessen the danger; but such means are obviously unreliable. I have known medical students in our several hospitals to have intercourse with the inmates, relying on a previous cauterization of chancres or mucous patches with the nitrate of silver, and yet contract syphilis. Possibly, the application of the carbo-sulphuric paste, which appears for a time to dry up the secretion of venereal ulcers, and which forms a very adherent scab, might have been more efficacious, but it is an experiment not likely to be tried except by a fool or a drunken man.

Three centuries ago, Nicholas Massa enunciated the precept: "*Si vero quis cum infecta muliere coire voluerit, quod fatuum est, non moretur in coitu.*" The longer the exposure, the greater the danger, not only because contact with the virus is prolonged, but also because the virulent secretion is constantly on the increase. Frequent repetition of the act is open to the same objection, and although the precept "*Non bis in idem*" may be too strict for the ardor of youth, "*Non quater in idem*" is both reasonable and prudent.

After exposure, the utmost attention is, for both sexes, most desirable. Although we have reason to believe that the absorption of the syphilitic virus, if it meets with any solution of continuity, is instantaneous, yet doubtless it often remains for some time upon the surface, until, after first acting as a common irritant, it gains entrance beneath the epidermis. In the latter case, there is still an opportunity to remove it by careful ablution, either with simple water, or preferably, with soap and water, water slightly acidulated with vinegar, or a weak solution of carbolic acid, taking care to pursue the enemy in the folds at the sides of the frænum and around the vulva, and in similar situations, where he is most prone to lurk.

There is a danger little dreamed of by persons indulging in illicit intercourse; I refer to that of contracting syphilis elsewhere than upon the genital organs, and especially upon the mouth. I have had several men under my care who have thus contracted chancres upon the lips, while their genital organs escaped contagion, and this, too, in the absence of any unnatural mode of indulgence. If this occurs with men, the danger must be still greater for women, since mucous patches are well known to be far more frequent in the male than in the female sex.

I do not propose at present to take up the prophylaxis of venereal diseases through the legal restriction of prostitution, but desire to make a single remark upon the subject. If the men who visit houses of ill-fame could be subjected to examination, far more would be done to suppress disease than by any visitation of the female inmates of such places, since with the former the evidences of health or disease are much more patent and more easily recognized than in women

This is already being done by the better class of prostitutes in Paris, who, to protect themselves from the confinement to which they are subjected in case they are discovered to be diseased, will not allow a man to cohabit with them unless they have first made an examination of his person, in which they become quite expert. Thus the police regulations adopted in that city are accomplishing their object in an unexpected way.

